# Assessment of Salt, Potassium, and Iodine Intake in the Croatian Adult Population Using 24 h Urinary Collection: The EH-UH 2 Study

**DOI:** 10.3390/nu16162599

**Published:** 2024-08-07

**Authors:** Mihaela Marinović Glavić, Lovorka Bilajac, Marta Bolješić, Marija Bubaš, Krunoslav Capak, Marija Domislović, Aleksandar Džakula, Mirjana Fuček, Lana Gellineo, Ana Jelaković, Josipa Josipović, Tomislav Jukić, Denis Juraga, Ivan Pećin, Vladimir Prelević, Danilo Radunović, Željko Reiner, Tomislav Rukavina, Petar Šušnjara, Vanja Vasiljev, Valentina Vidranski, Bojan Jelaković

**Affiliations:** 1 Department of Social Medicine and Epidemiology, Faculty of Medicine, University of Rijeka, 51000 Rijeka, Croatia; lobilajac@gmail.com (L.B.); ana.jelakovic9@gmail.com (A.J.); denis.juraga@uniri.hr (D.J.); tomislav.rukavina@uniri.hr (T.R.); vanjav@uniri.hr (V.V.); 2 Department of Public Health, Faculty of Health Studies, University of Rijeka, 51000 Rijeka, Croatia; 3 Teaching Institute of Public Health Primorje—Gorski Kotar County, 51000 Rijeka, Croatia; 4 Department of Anatomy and Neuroscience, Faculty of Medicine, University of Osijek, 31000 Osijek, Croatia; boljesic1997@gmail.com; 5 Croatian Institute of Public Health, 10000 Zagreb, Croatia; marija.bubas@miz.hr (M.B.); kcapak@hzjz.hr (K.C.); 6 Ministry of Health, 10000 Zagreb, Croatia; 7 Department for Nephrology, Hypertension, Dialysis and Transplantation University Hospital Centre, 10000 Zagreb, Croatia; marija.domislovic@kbc-zagreb.hr (M.D.); lana.gellineo@gmail.com (L.G.); vladimir.scopheuope@gmail.com (V.P.); danilo.radunovic@kccg.me (D.R.); 8 School of Medicine, University of Zagreb, 10000 Zagreb, Croatia; tomislav.jukic@kbcsm.hr (T.J.); ivanpecin@yahoo.com (I.P.); 9 Department of Social Medicine and Organization of Health Care, Andrija Štampar School of Public Health, University of Zagreb School of Medicine, 10000 Zagreb, Croatia; adzakula@snz.hr; 10 Department of Laboratory Diagnostics, University Hospital Centre Zagreb, 10000 Zagreb, Croatia; mirjana.fucek@kbc-zagreb.hr; 11 Department of Nephrology and Dialysis, Sestre Milosrdnice University Hospital Centre, 10000 Zagreb, Croatia; josipa.josipovic01@gmail.com; 12 School of Medicine, Catholic University of Croatia, 10000 Zagreb, Croatia; 13 Department of Oncology and Nuclear Medicine, Sestre Milosrdnice University Hospital Centre, 10000 Zagreb, Croatia; 14 Department for Metabolic Diseases, University Hospital Centre Zagreb, 10000 Zagreb, Croatia; zreiner@kbc-zagreb.hr; 15 Clinic for Nephrology, Clinical Centre of Montenegro, 81000 Podgorica, Montenegro; 16 Department of Cardiology and Congenital Diseases of Adults, Polish Mother’s Memorial Hospital Research Institute, 93-338 Lodz, Poland; 17 Faculty of Kinesiology Osijek, Josip Juraj Strosssmayer, University of Osijek, 31000 Osijek, Croatia; psusnjara1@gmail.com; 18 Department of Clinical Chemistry, Sestre Milosrdnice University Hospital Centre, 10000 Zagreb, Croatia; vvidranski@gmail.com; 19 Croatian Hypertension League, 10000 Zagreb, Croatia

**Keywords:** salt, sodium, potassium, iodine, 24 h urine, population-based study

## Abstract

Cardiovascular diseases, which are the leading cause of death in Croatia, are linked to the high prevalence of hypertension. Both are associated with high salt intake, which was determined almost two decades ago when Croatian Action on Salt and Health (CRASH) was launched. The main objective of the present study was to evaluate salt, potassium, and iodine intake using a single 24 h urine sample in a random sample of the adult Croatian population and to analyse trends in salt consumption after the CRASH was intensively started. Methods: In this study, we analysed data on 1067 adult participants (mean age 57.12 (SD 13.9), men 35%). Results: Mean salt and potassium intakes were 8.6 g/day (IQR 6.2–11.2) and 2.8 g/day (IQR 2.1–3.5), respectively, with a sodium-to-potassium ratio of 2.6 (IQR 1.8–3.3). We detected a decrease of 17.6% (2 g/day less) in salt consumption compared with our previous salt-mapping study. However, only 13.7% and 8.9% met the WHO salt and potassium recommended targets of 5 g/day and 3.5 g/day, respectively. Salt intake was higher, and potassium ingestion was lower, in rural vs. urban regions and in continental vs. Mediterranean parts of Croatia. Moderate to severe iodine insufficiency was determined in only 3% of the adult participants. Conclusion: In the last fifteen years, salt consumption has been significantly reduced in the Croatian adult population because of the intensive and broad CRASH program. However, salt intake is still too high, and potassium ingestion is too low. Salt reduction programs are the most cost-effective methods of cardiovascular disease prevention and merit greater consideration by the government and health policy makers.

## 1. Introduction

### 1.1. Global Burden of Salt and World Health Organization Recommendations

According to the World Health Organization (WHO) Global Report on Hypertension, high blood pressure (BP) is the main risk factor for early death worldwide [1]. High salt consumption was the major cause of hypertension and more than 3 million deaths, and 70 million disability-adjusted life years (DALYs) globally are attributed to high sodium intake [2]. The latest Global Burden Disease Study (GBDS) report states that high sodium consumption was responsible for 1.72 million deaths and 40.54 million DALYs due to cardiovascular disease (CVD), an increase of 41.08% and 33.06%, respectively, compared with 1990 [3]. The total number of deaths from chronic kidney disease (CKD) associated with increased salt intake was 45,530, while the number of DALYs was 1.32 million [4]. It was also confirmed that high salt intake increases the risk of type 2 diabetes [5]. In 2013, the WHO recommended that all Member States reduce population salt intake by 30% as a part of the nine global voluntary targets to reduce premature mortality from chronic non-communicable diseases (NCDs) by 25% by 2025 [6]. Since reducing salt intake is the most cost-effective intervention to reduce the burden of NCDs, it is considered a priority action for all countries [7,8,9,10]. Recommendations for daily potassium intake vary among institutions, and the WHO recommends at least 3510 mg/day, which is in line with the European Food and Safety Authority’s recommendations of an adequate intake of 3500 mg for both sexes [11,12,13].

The average daily potassium intake worldwide is around 2250 mg, which is below all the recommended values [14]. Progressively, more studies indicate that the sodium-to-potassium ratio independently predicts the upcoming risk of CVD events, and the WHO recommended a sodium-to-potassium ratio ≤ 1 [15,16].

### 1.2. Cardiovascular Disease Burden in Croatia and Croatian Action on Salt and Health

In 2022, CVDs were the main cause of death in Croatia (39.1%), and the age-standardized death rate in Croatia was significantly higher than the EU average (591/100,000 vs. 344/100,000) [17]. Ischemic heart disease was the leading cause of death (12.2% or 6925 deaths), and hypertensive disease was in second place (9.2% or 5231 deaths), which indicates that hypertension is the main cause of death in Croatia. High BP ranks second after smoking as an important risk factor for DALYs in Croatia [18]. The current crude prevalence of hypertension in Croatia is very high (50.9%), which is higher than 15 years ago [19,20].

In a recent study published in 2023, the authors estimated that men from Croatia will have the highest age-adjusted prevalence of hypertension worldwide in 2040, reaching 41.1% [21].

In a salt-mapping study organized in 2005, the average intake of salt determined from sodium in 24 h urine was 11.3 g per day (men 13.37 g, women 10.37 g) [22,23].

These results are like the results obtained in other European countries, particularly in neighbouring Slovenia and Montenegro (REF WHO report Europe). Being aware of this result, a Declaration on the importance of starting a national campaign to reduce salt intake in Croatia was accepted at the Congress of the Croatian Society for Hypertension in 2006, and in 2007, the Croatian Action on Salt and Health (CRASH) program started [22,24,25,26,27,28]. The CRASH program was prepared according to the WHO recommendations [29]. CRASH consists of educating the population, healthcare workers, and patients, aiming to increase awareness of the harmful effects of excessive salt intake, and, importantly, negotiating with the food industry, aiming to reformulate recipes. In 2015, the Ministry of Agriculture adopted the Ordinance on Cereals and Cereal Products, which determined that the proportion of salt in ready-to-eat baked bread should not exceed 1.4%. In 2022, as the second step, it determined that the salt content of ready-to-eat baked bread, as well as baked pastry, should not exceed 1.3% [23,30].

From 2010 to 2019, the intake of salt from eating bread and bakery decreased by about 14% and 28%, respectively, and most of the bakery industry was found to be compliant with the regulation (72% of bread and 66% of bakery products had a salt content <1.4%) [23,31]. The biggest Croatian meat industry, PIK Vrbovec, decreased the content of salt in all products by an average of 25% [23]. The objective of our study was to estimate salt, potassium, and iodine intake in a random sample of the Croatian adult population by evaluating sodium, potassium, and iodine excretion in 24 h urine samples. It is our wish and hope that the obtained results will enhance the activities of government and health policy makers.

## 2. Materials and Methods

### 2.1. Study Design and Recruitment

This cross-sectional study was part of the EH-UH 2 survey (epidemiology of arterial hypertension and salt intake in Croatia), a nationally representative survey of noninstitutionalized persons in Croatia, which involved collecting anthropometric, demographic, lifestyle, and biological marker data from the general adult population to assess risk factors for cardio–kidney–metabolic health and the prevalence of major NCDs. A total of 2021 participants were included by random selection from the general population using randomization numbers obtained from the registries of family physicians. The randomization numbers represented the ordinal numbers of the archive of each family physician’s practice, and they were the personal ID codes of each subject, which also enabled planned follow-up. Outpatient examinations were organized on Fridays and Saturdays; thus, a single 24 h urine sample was collected during a regular working day (Thursday or Friday) to avoid family/friend meetings and eating (lunch, dinner, barbecue); examinations were not performed during festive seasons. This study was carried out in three steps as follows: (a) questionnaire survey, (b) physical measurements, and (c) blood drawn, spot urine samples, and 24 h urine collections. Nurses, physicians, pharmacists, fellows, residents, and medical students who were members of the mobile examination team (MET) were educated to collect study and clinical data in a standardized manner. More details can be found in the Supplementary Materials.

### 2.2. Procedures of 24 h Urine Collection

**Figure 1:**
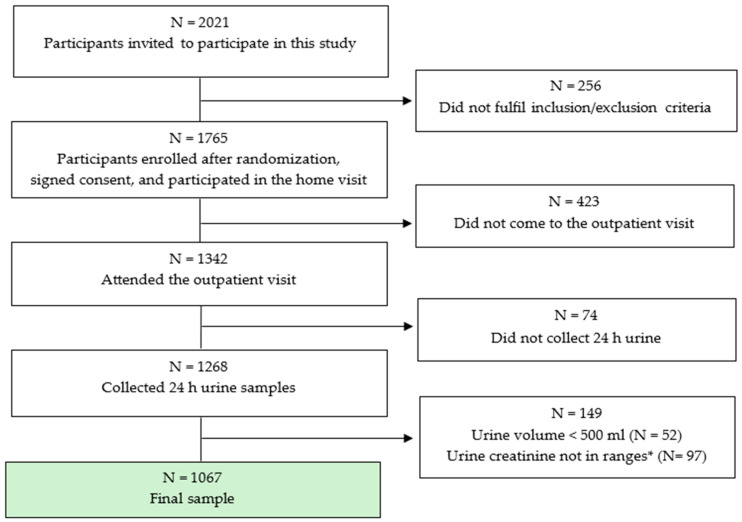
Final number of participants included in this study. * Ranges for adequate urine creatinine (5.9–26.0 mmol/24 h for men; 4.0–16.4 mmol/24 h for women).

Participants were provided with 2.5 L plastic polyethylene containers, and a clear verbal explanation was given as well as detailed written instructions on how to collect the 24 h urine sample, emphasizing the importance of collecting every drop of urine. They were asked to discard the first urine voided on the day of their collection and to collect all urine voided during the subsequent 24 h period, ending with the first urine void the following morning. The day before the outpatient visit, the participants were reminded by the phone call on how to properly collect the 24 h urine sample. During the outpatient visit, the MET staff measured and recorded the 24 h urine volume, thoroughly mixed the collection, and retained three 2 mL aliquots, which were stored at 4 °C and shipped the same day. The first aliquot was shipped to the Central Laboratory in University Hospital Centre Zagreb, where urine creatinine, potassium and sodium were analysed; the second aliquot for iodine analyses was sent to the laboratory of University Hospital Centre Sestre milosrdnice, Zagreb. The third aliquot was left as a backup, if needed. The total volume of urine collected was measured using a measuring cylinder. Quality assurance to avoid inclusion of incomplete urine collections (under-/over-collected) was applied, and 24 h urine specimens were considered incomplete if (i) the start and end times were not recorded and could not be ascertained; (ii) the length of the collection time was out of the range of 22–26 h; (iii) the total urine volume was less than 500 mL; (iv) a female participant was menstruating; and (v) a participant reported that more than a few drops of urine were missed during collection. Further predefined criteria for a high likelihood of incomplete urine collection were values of urine creatinine content versus body weight outside 2 standard deviations of the sex-specific distribution (5.9–26.0 mmol/24 h for men and 4.0–16.4 mmol/24 h for women). Figure 1 shows the stepwise procedure for the selection of valid participants according to protocol adherence, quality control, and completeness of the 24 h urine collections. For iodine status analysis, another 119 subjects with thyroid disorders or known consumption of iodine supplements were also excluded. More details on the methods for sodium, potassium, and iodine determination can be found in the Supplementary Materials [32,33,34,35,36,37,38].

### 2.3. Statistical Analysis

According to the WHO/PAHO Regional Expert Group for Cardiovascular Disease, to detect an approximately 1 g reduction in salt intake over time using 24 h urinary sodium excretion (difference~20 mmol/24 h), with a standard deviation of 75 mmol/day (alpha = 0.05, power = 0.80), a minimum sample of 120 individuals per stratum is recommended [35]. Thus, a minimum recommended sample size of 170 was estimated per age and sex group and adjusted for an anticipated non-response rate of 30% (this was the non-response rate in our previous survey). The population was stratified into groups by sex (men and women), age (A. 18–29 years; B. 30–44 years; C. 45–59 years; D. 60–75 years), and urban/rural and Mediterranean/continental areas. Finally, 1942 individuals were needed to be selected (total *n* = 170 × 8 groups/0.7 attrition = 1942). In relation to the geographical regions of the participants’ places of residence in this survey, we divided the group into five different regions of Croatia as follows: the Central part (continental), the North–West part (continental), Slavonia (continental), Istria, Croatian Littoral, the Gorski Kotar region (Mediterranean mountain), and Dalmatia (Mediterranean) According to the WHO recommendation, the prevalence of 24 h sodium excretion lower than 85 mmol (corresponding to 5 g of salt) per day was calculated. All statistical analyses were carried out with IBM SPSS Statistics 28.0.0.0. (IBM Corporation, Armonk, NY, USA). For continuous variables, after testing for a normal distribution (Kolmogorov–Smirnov and Shapiro–Wilk tests), either a Student’s *t*-test for unpaired samples, an analysis of variance (ANOVA), or the Mann–Whitney test (for non-normally distributed results) was used to assess differences among group means, and the Pearson chi-square test was performed to determine associations among categorical variables. The results were reported as mean (standard deviation—SD), median (interquartile range—IQR), or percentages, as appropriate. Pearson and Spearman correlation coefficients were calculated to obtain information about the relationships among variables.

The survey was carried out in accordance with the Declaration of Helsinki and Good Clinical Practice [39]. Ethical approval for the survey was obtained from the Ethics Committee of the School of Medicine, University of Zagreb, and the participants were provided a written informed consent to sign.

## 3. Results

After the exclusions reported above, the final nationwide population sample included 1067 participants between 18 and 89, recruited from five Croatian regions (Central part, North–East part—Slavonia, North–West part, North part of the coast, and South part of the coast—Dalmatia).

### 3.1. Completion of 24 h Urine Collection

From 2018 to 2021, 1765 adults aged 18 to 91 years were randomized and participated in the examination component of the EH—UH 2 survey (Figure 1). Among these 1765 participants, 1342 (76%) attended the outpatient visit and were asked to participate in a 24 h urine collection examination. Overall, 1268 (94.5%) participants (35% men) completed a 24 h urine collection, with 1067 (84.1%) participants collecting an adequate urine sample. These participants were included in further analyses. There were no differences between the group of subjects that participated in a 24 h urine collection examination and those who refused to participate in age, BMI, or diastolic BP, but in the group that did not collect 24 h urine samples, there were more men (44% vs. 35%), and they had lower systolic BP (129.9 vs. 133.6 mmHg). The response rate of the enrolled subjects for the 24 h urine collection was estimated at approximately 72% ((94.5% (24 h urine completion rate) × 76% (attended the EH-UH 2 outpatient visit)), and adequate 24 h urine collection was estimated at approximatively 63.8% (84.1% adequate 24 h urine sample) × 76% (attended the EH-UH 2 outpatient visit)). The overall survey response rate for the 24 h urine collection was estimated at approximately 69% (94.5 (24 h urine completion rate) × 73% (EH-UH 2 response rate)), and the adequate survey 24 h urine collection was 61% (84.1% (24 h urine completion rate) × 73% (EH-UH 2 response rate)).

### 3.2. Characteristics of the Participants Who Attended the Outpatient Visit and Were Asked to Participate in a 24 h Urine Examination

Table 1 shows the characteristics of the participants. The average age of the cohort was 57.2, which is higher than the average age of the general Croatian adult population of 44.3 (men 42.5, women 45.9), according to the 2021 census [40]. A higher average age and a lower proportion of men in the sample compared with the general population is a result of a better response rate among the older population and women. Average BP values were in high-normal BP ranges, and the average BMI value was in the overweight category. There were no statistically significant differences in the mean age and heart rate between men and women; however, men were taller and heavier with higher BMIs and higher values of systolic and diastolic BP.

### 3.3. Daily Urinary Excretions of Creatinine, Sodium, Potassium, and Iodine and Estimated Salt, Potassium, and Iodine Intake in the Whole Group

**Figure 2:**
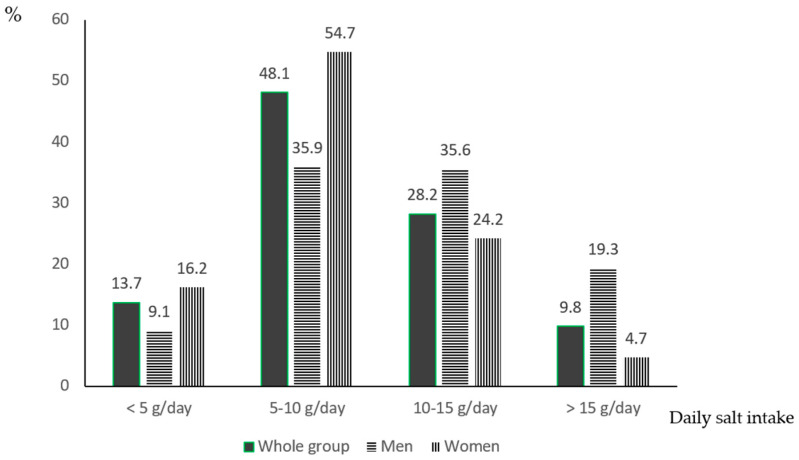
Distribution of single 24 h salt intake estimates.

Results of the analyses of the 24 h urine sample are presented in Table 2. Average urinary volume excretion was 1589.9 mL/day, being higher in men than in women. Mean urinary sodium excretion was 140.5 mmol/24 h (IQR 101.5 g–191.3 g), corresponding to a mean consumption of 8.6 g of salt per day (IQR 6.2 g–11.7 g). We observed a significant difference between men and women in daily salt consumption, which was significantly higher in men than in women (10.5 g (IQR 7.3 g–14.0 g) vs. 8.0 g (IQR 5.8 g–10.5 g), *p* < 0.001), equivalent to ~2.5 g of higher salt consumption by men than women. Only 13.7% of the population met the WHO criteria of less than 5 g of salt per day (Figure 2). Significantly more women than men ingested the recommended amount of salt per day (16.2% vs. 9.1%; χ2 = 10.404; *p* = 0.0012). Men consumed more potassium than women (3.1 g per day (IQR 2.3 g–3.9 g) vs. 2.6 g per day (IQR 2.0 g–3.3 g); *p* < 0.001)), and more men than women met the WHO recommended levels of potassium intake of 90 mmol/day or more (χ2 = 17.5; *p* = 0.000002). The higher salt and potassium intake observed in men could be explained by the fact that men on average eat more food than women. Other social and behavioural factors that contribute to these differences will be further analysed and discussed in our forthcoming paper. We observed a significant correlation between salt and potassium consumption in both men and women (R = 0.223 and R = 0.291, respectively; *p* < 0.001 for both) (Figures S1–S3). In the whole group, only 3.7% of the subjects had a salt-to-potassium ratio of less than 1. Interestingly, women had a lower sodium-to-potassium ratio than men (2.5 IQR 1.8–3.3 vs. 2.8 IQR 1.9–3.8; *p* < 0.001). Although more women than men had a ratio of less than 1, the difference was not significant (4.2% vs. 2.9%; χ2 = 1.06; *p* = 0.303). In the whole group, the overall median of urinary iodine excretion was in the recommended range (131.8 µmol/L, IQR 95.4–174), indicating adequate iodine intake. Men excreted significantly more iodine than women (*p* < 0.001), which is related to the higher salt intake in men. We found a significant correlation between salt and iodine intake in the whole group (R = 0.52; *p* < 0.001) as well as in men (R = 0.57; *p* < 0.001) and women (R = 0.45; *p* < 0.001) (Figures S4–S6). In the whole group, 22.3% of the subjects had urinary iodine excretion below 100 µg/L (Table 3). However, most of the subjects (70.9%) had urinary iodine excretion in the “adequate” and “above requirement” categories (Table 3). However, the majority had mild insufficiency (86%). There were no differences between men and women in urinary iodine excretion values below 100 µg/L (χ2 = 1.889; *p* = 0.203). Although more men than women were in iodine categories “above requirement” (31.7% vs. 22.2%), we failed to find a statistically significant difference (χ2 = 3.243; *p* = 0.07). Excessive iodine intake with urinary iodine excretion values ≥300 µg/L was found in 6.8% of participants.

### 3.4. Daily Salt, Potassium, and Iodine Intake according to Residency and Geographic Regions

**Figure 3:**
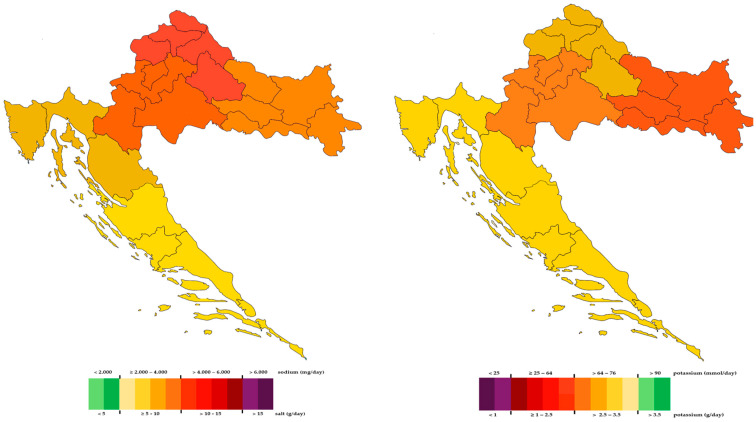
Observed median 24 h urinary sodium/salt (**left**) and potassium (**right**) excretion by region. Red (green) indicates a high (low) level of 24 h urinary sodium (**left**) and potassium (**right**) excretion.

**Figure 4:**
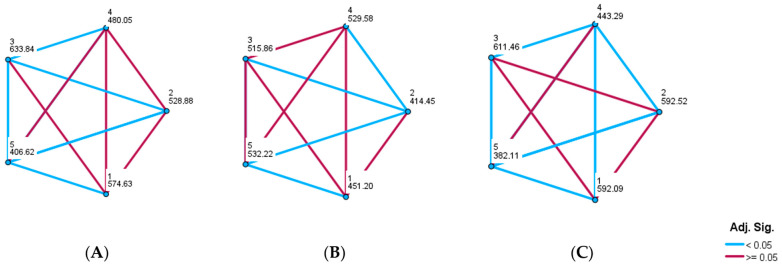
Pairwise comparisons of daily salt (**A**) and potassium (**B**) intake and the sodium-to-potassium ratio (**C**) among five Croatian regions. Each node shows the sample average rank of five different regions; 1 = Central part; 2 = North–East—Slavonia; 3 = North–West part; 4 = North part of the coast; 5 = South part of the coast—Dalmatia.

Results of salt, potassium, and iodine consumption according to residency are presented in Table 4. Salt intake was significantly higher, while potassium intake was significantly lower, in rural vs. urban areas (*p* < 0.001; *p* = 0.006, respectively). When we analysed geographical variability, we found that salt intake was significantly lower and potassium intake was significantly higher in the Mediterranean part of Croatia compared with the continental part (*p* < 0.001 for both). The sodium-to-potassium ratio was significantly lower in urban vs. rural areas, and in the Mediterranean vs. the continental part of Croatia (*p* < 0.001 for both). As shown in Table 5 and Figure 3 and Figure 4, daily salt intake was significantly lowest in the southern part of the Croatian coast, in Dalmatia (7.37 g) compared with all other regions including the northern part of the Croatian coast (8.46 g). However, salt intake in the northern part of the Croatian coast was lower than salt intake in all three continental regions of Croatia. The highest salt intake was observed in the North–West part of Croatia (11.0 g) compared with all other regions. Daily potassium intake was significantly higher in both Dalmatia (3.0 g) and the northern part of the Croatian coast (3.0 g) than in all other continental regions. The lowest potassium intake was found in the North–East part of Croatia, in Slavonia (2.4 g). The sodium-to-potassium ratio was significantly lower in both Dalmatia and the northern part of the Croatian coast than in all other continental regions. The highest sodium-to-potassium ratio was observed in the North–West part of Croatia. There were no significant differences among the continental parts of Croatia in either potassium ingestion or the sodium-to-potassium ratio. Regional dietary habits, demographic, and socioeconomic factors that could contribute to the observed differences among regions will be further analysed and reported in our forthcoming paper.

## 4. Discussion

The EH-UH 2 study is the first nationwide study on salt, potassium, and iodine consumption in the Croatian general adult population. The results showed that in both genders, salt consumption was still high and potassium intake was too low. In the majority of subjects, iodine excretion was in the recommended ranges, and only in a very small proportion of the population, it was severely insufficient.

### 4.1. Salt Consumption in the General Adult Population

The average population salt consumption in our adult sample of 8.6 g/day was above the WHO recommended value of 5 g/day. However, in a systematic review of population-level intake in the WHO European region in only a few countries (Norway, the Netherlands, Italy, and the United Kingdom), the mean salt intake was below 9 g/day [41]. In the EH-UH 2 study, salt intake was significantly higher in men (10.5 g/day) than in women (8.0 g/day), which was in concordance with reports from most countries [41]. The Croatian data, which were included in this study, were collected in our previous salt-mapping study conducted in 2005. The same methodology was used in the EH-UH 2 study, and salt ingestion at the population level was 11.3 g/day (men 13.4 g/day, women 10.4 g/day) [22]. The significant improvement at the population level found in the EH-UH 2 study (2 g/day less, or a 17.6% decrease in salt consumption with no gender difference) is a result of the broad and intensive CRASH program, which started in 2006 [23]. As already mentioned, the CRASH program consists of educating the general population, patients, and healthcare workers about the harmful effects of high salt consumption (using various channels for communication) and negotiating intensively with the food industry.

In 2021, Santos et al. published a systematic review of national salt reduction initiatives. Croatia was not included in their report, while at that time, the results of our nationwide EH-UH 2 study were not available [42]. Only fourteen countries out of the 96 included in their report analysed data on program effects, and among them, 9 reported a moderate decrease (1–2 g/day), 3 reported a substantial decrease (>2 g/day), and 5 reported a slight decrease (<1 g/day). It seems that success mostly depends on the engagement and enthusiasm of non-governmental organizations and the willingness of the food industry to participate. This was the case in Croatia, where most of the work was performed by the Croatian Society of Hypertension and the Croatian Hypertension League with the help of the Croatian Society of Atherosclerosis. We have established an excellent collaboration with the meat industry. Moreover, an important contribution was made by the Ministry of Agriculture, which prepared an ordinance for the bakery industry that limits salt content in final bakery products to no more than 1.3%. The Croatian Institute of Public Health and the Croatian Agency for Agriculture and Food participated and strongly supported all these activities. In the EH-UH 2 study, the WHO target of 5 g/day was found in only 13.7% of the population (9.1% men, 16.2% women). This is significantly better than 3.7%, which was registered in our salt-mapping study 15 years ago, but far away from the main WHO aim. Similar results of 11.3% and 12.5% were found in Moldova and Lithuania, respectively [43,44]. Montenegro, Hungary, and Greece reported that 7%, 6.9% and 5% of the population reached the WHO target, respectively [45,46]. After an average of 5 years of the most intensive CRASH activities, we achieved a 3.5% decrease in salt consumption per year at the population level. A decrease of 4% per year was achieved only in Italy and North Karelia. The result that only 13.7% of our population reached the WHO target of 5 g/day is very concerning and should motivate government and health policy makers to become more engaged and active.

### 4.2. Potassium Consumption in the Adult Population

It is well known that low potassium intake is related to higher BP and CVD risks, and vice versa [47]. Thus, the WHO recommended that potassium intake should be at least 3.5 g/day for both genders [12]. According to our results, the average potassium intake in the Croatian adult population of 2.9 g/day is significantly below this target, but it is above the average word intake of 2.3 g/day [14]. Men ingested more potassium than women, and this could be explained, similar to salt consumption, by the fact that men eat more food than women. Our result agrees with the results obtained in Hungary, but the average amount was lower than the results published for Montenegro, Moldova, Greece, and Lithuania. In our cohort, only 14.7% of the adult population reached the WHO target of at least 3.5 g/day (90 mmol/day), which is in concordance with the results reported from Montenegro but lower than the results found in Lithuania, Hungary, Greece, and Moldova [43,44,45,46,48].

### 4.3. Sodium-to-Potassium Ratio

In our cohort, the average sodium-to-potassium ratio was 2.6, which was lower than that found in Hungary [45] and in the INTERSALT study [49] but higher than that found in Greece and Lithuania [14,46]. We found the sodium-to-potassium ratio to be lower in women than in men, which was the case in Norway, the NHANES study, Finland, New Zealand, Italy, Hungary, Greece, and Lithuania [44,46,50,51,52,53]. Besides Greece, where the ratio was closest to one, in the vast majority of countries, the sodium-to-potassium ratio was reported to be significantly higher than recommended, reflecting globally unhealthy eating habits with too much salt and not enough vegetables and fruits.

### 4.4. Salt and Potassium Intake according to the Residency and Geographical Differences

Salt intake and the sodium-to-potassium ratio were higher, and potassium intake was lower, in rural than urban areas, indicating that eating habits were worse among rural populations. In studies conducted in Canada and Moldova, sodium and potassium intake were higher in rural than urban regions, but the sodium-to-potassium ratio was slightly better in urban regions [43,54]. It seems that our rural population eats fewer vegetables and fruits than farmers in Canada and Moldova. This should be taken into consideration not only for educational plans but also for activities and national programs, which should be adjusted when organized by the government. Croatia could be separated into two regions, i.e., continental and Mediterranean, which differ in geography but even more importantly, in history, culture, gastronomy, and lifestyle. Differences between Dalmatia (Mediterranean part) and Slavonia (continental part) were reported for the first time in 1966 in the Seven-Country Study, where it was observed that the population living in Dalmatia had lower BP with less obesity than the population from Slavonia [55]. Croatia is an excellent model to test and analyse, using the same population, whether the Mediterranean style of living is still present. We found salt intake and the sodium-to-potassium ratio to be lower, and potassium intake to be higher, in the Mediterranean part than in the continental part. Although these results indicated that eating habits concerning salt and potassium intake were better in the Mediterranean part, the average salt consumption was still too high, and the average consumption of potassium was still too low. We further analysed differences in salt and potassium intake among five different Croatian regions—two from the coast and three from continental regions. The lowest sodium intake and sodium-to-potassium ratio and the highest potassium intake found in both Mediterranean parts of Croatia could be explained by still preserved traces of the Mediterranean way of living. Finding no differences in the high sodium-to-potassium ratio among continental regions reflects equally poor eating habits in continental Croatia, with too salty food and insufficient consumption of vegetables and fruits. In the MINISAL-GIRSCI programme, the authors found a significant north–south pattern of sodium excretion in Italy [56]. These spatial differences and geographical variations were partly explained by a socioeconomic gradient. In our forthcoming paper, we plan to determine which factors and variables could most plausibly explain geographical differences in Croatia.

### 4.5. Iodine Intake in the Adult Population

The relationship between iodine excretion and salt intake varies based on the iodine content in salt and dietary habits [57,58]. By comparing iodine intake between the group that achieved the goal of salt intake (less than 5 g per day) and the group that did not achieve the recommended salt intake, we found a significant difference (115.1 (72.6–171.) vs. 220.4 (163.2–298.0); *p* < 0.001). However, the overall median of urinary iodine excretion was in the recommended range, indicating adequate iodine intake. In the whole group, slightly more than 20% of the subjects had urinary iodine excretion below the recommended values, and most of the subjects had urinary iodine excretion in the “adequate” and “above requirement” categories, which indicates that for most of the population, the overall median of urinary iodine excretion falls within the recommended range. Our results are in line with most of the countries that have well-established iodine fortification programs, particularly iodized salt, leading to generally adequate iodine intake [59]. Moderate to severe iodine insufficiency was determined in only 3% of the adult participants, indicating that further lowering salt consumption will not endanger the majority of the population. However, because of the fear of iodine insufficiency, in accordance with Croatian legislation, the amount of iodine per kilogram of salt can be increased if necessary [60,61].

## 5. Strengths and Limitations

According to our knowledge, this is the first study on salt, potassium, and, at the same time, daily iodine excretion in Croatia. The strengths of our population-based study are (i) the enrolment of a large random sample of men and women in the Croatian adult population from different parts of the country; (ii) salt and potassium intakes were measured using the gold-standard method of 24 h urine collections; (iii) a rigorous quality control was performed, and a highly standardized protocol was used, enabling us to include only valid urine samples into the analyses; and (iv) the participation rate was very good, and there were no substantial differences between the group included in analyses and the group of subjects who either refused to participate or the group of subjects whose urine samples were inadequate. The limitations of our study are (i) urinary sodium, potassium, and iodine were only assessed once and (ii) we did not use PABA to determine the completeness of urine collection.

Although a 24 h urine sampling is considered a golden standard for estimating salt and potassium intake, it can result in falsely low (under-collection of urine) or falsely high (overcollection of urine) results. The debate on the accuracy and usefulness of this method is still ongoing [62]. The International Consortium for Quality Research on Dietary Sodium/Salt (TRUE) recommended assessing the population’s current 24 h dietary sodium ingestion using single complete 24 h urine samples collected over a series of days from a representative population sample [63]. The vast majority of studies, including the INTERSALT and INTERMAP studies, analysed single 24 h urine samples; thus, our results could be compared with them [64,65]. Furthermore, in the INTERSALT study, neither urine creatinine nor PABA was used to assess the completeness of urine collection. In the TRUE position statement, the authors concluded that assessing average population sodium intake is minimally affected by the random component of day-to-day variation in sodium excretion in individuals because the random over- and under-estimates of individual sodium intake are balanced when calculating the population average. They highlighted that the greatest caveats are (a) the completeness of 24 h urine collections; (b) urine should be collected on days that are representative of the usual population pattern of sodium intake (e.g., a mixture of weekends and weekdays); (c) the participants need to be representative of the population in question; and (d) seasonal (agricultural or climatic influences) or cultural variations in dietary patterns should be taken into account. As mentioned before, in our study, (a) we conducted quality control to assess the completeness of urine sampling; (b) the urine samples were collected on Thursday and Friday but not on Saturday or Sunday; (c) the participants were enrolled from all parts of Croatia, and the number of participants enabled us to make reliable conclusions; and (d) outpatient visits and field work were organized throughout the whole year besides August.

## 6. Actionable Recommendations and Policy Suggestions

Our results can provide the government and the Ministry of Health with firm evidence-based facts that strongly support CRASH activities, thus suggesting that a salt reduction program should be one of the priorities for the prevention of NCDs, particularly CVD. The government should (i) promote a salt reduction program by supporting our visibility in various media outlets (TV, radio, newspapers); (ii) prepare a plan for taxation, such as decreased taxation for products with lower salt content and low-salt substitutes and increased taxation for those who are not cooperative; (iii) be active in negotiations with the food industry for changing recipes. After we finish our forthcoming paper, which will present the variables that affect salt and potassium intake in different parts of Croatia, public health campaigns could be more specifically tailored to different geographical regions. They also should support and participate in our digital education programs.

## 7. Research Questions and Plans for Further Studies

After this initial overall report on salt, potassium, and iodine intake in the Croatian adult population, we plan to complete the analyses and studies listed in Table 6.

## 8. Conclusions

In the last fifteen years, salt consumption has been significantly reduced in the Croatian adult population. The reduction was undoubtedly facilitated by the intensive and broad CRASH program led by the Croatian Society of Hypertension and the Croatian Hypertension League, in which many public awareness campaigns (including a digital technology educative website [67]) have increased health literacy and helped to broader health trends and improve hypertension control [68]. One of the most important achievements of the CRASH program is the partnership and collaboration with the food industry, which changed recipes and practices. However, salt intake is still too high, and potassium ingestion is too low. Only 13.7% of the population reached the WHO target of 5 g of salt per day, and only 8.9% of the population ingested at least 3.5 g of potassium per day. The poorer sodium-to-potassium ratio observed in rural areas should be considered when future national programs are planned. The better results found in the Mediterranean part might reflect the still present rudiments of the Mediterranean way of living. However, even in the Mediterranean part of Croatia, salt intake is still too high. Moderate to severe iodine insufficiency was determined in only 3% of the adult participants, indicating that further lowering salt consumption will not endanger the majority of the population. However, if needed, according to the Croatian legal regulation of the mandatory iodization of all types of salt, the amount of added iodine can be easily increased from the current 15–23 mg of iodine per kilogram of salt [60,61,69,70,71,72].

The CRASH program achieved a very good result, which more than proves its feasibility. With the expected more active engagement and help from the government, the obtained results could be significantly accelerated and improved. Salt reduction programs are among the most cost-effective methods of CVD prevention and merit greater consideration by the government and health policy makers [73].

## Figures and Tables

**Table 1:** Characteristics of the participants.

	Whole Group	Men	Women	*p*
N	1067	374	693	
Age (years)				
Mean (SD)	57.2 (13.9)	57.2 (13.8)	56.4	0.352
S.E.	0.37	0.75	0.55	
Median (IQ range)	59.0 (48.0–67.0)	59.0 (48–68)	59.0 (48–66)	
Systolic BP (mmHg)				
Mean (SD)	133.7 (19.0)	137.9 (18.3)	131.4 (19.2)	<0.001
S.E.	0.5	0.99	0.77	
Median (IQ range)	132 (120.0–145.0)	137.0 (124.5–149.0)	130.0 (117.0–133.8)	
Diastolic BP (mmHg)				
Mean (SD)	82.5 (10.2)	83.9 (10.2)	81.5 (10.0)	<0.001
S.E.	0.2	0.55	0.4	
Median (IQ range)	82.0 (76.0–89.0)	84.0 (77.5–90.5)	81.0 (75.1–87.5)	
Heart rate (bpm)				
Mean (SD)	75.7 (12.2)	75.1 (13.0)	76.4 (12.0)	0.084
S.E.	0.3	0.71	0.48	
Median (IQ range)	75.0 (67.0–83.0)	73.0 (65-0-83.3)	75.6 (68.0–83.0)	
Height (cm)				
Mean (SD)	169.1 (16.7)	178.4 (7.7)	165.3 (6.8)	<0.001
S.E.	0.4	0.42	0.27	
Median (IQ range)	168 (162–175)	178.0 (174.0–183.0)	165.0 (161.0–170.0)	
Weight (kg)				
Mean (SD)	81.4 (16.7)	92.4 (16.1)	76.6 (14.5)	<0.001
S.E.	0.4	0.88	0.58	
Median (IQ range)	80.0 (69.0–91.0)	90.0 (82.0–100.7)	75.5 (66.0–85.8)	
Body mass index (kg/m^2^)				
Mean (SD)	28.3 (5.0)	28.0 (4.6)	28.0 (5.3)	<0.001
S.E.	0.1	0.25	0.21	
Median (IQ range)	27.9 (24.8–31.2)	28.4 (26.0–31,4)	27.4 (24.0–31.1)	

**Table 2:** Daily urinary excretions of sodium, potassium, and creatinine and estimates of salt, potassium, and iodine intake.

24 h Urine	Whole Group	Men	Women	*p*
N	1067	374	693	
Volume (mL)				
Mean (SD)	1589.9 (628.5)	1670 (613.2)	1546 (632.9)	<0.001
S.E.	19.24	31.7	24.0	
Median (IQ range)	1500 (1100–1950)	1590 (1215–2000)	1500 (1100–1900)	
Creatinine (g/L)				
Mean (SD)	0.8 (0.4)	1.0 (0.5)	0.8 (0.4)	<0.001
S.E.	0.01	0.02	0.01	
Median (IQ range)	0.7 (0.5–0.1)	0.9 (0.6–1.3)	0.7 (0.5–0.9)	
Sodium (mmol/24 h urine)				
Mean (SD)	151.8 (68.8)	179.1 (74.9)	137.1 (60.4)	<0.001
S.E.	2.14	3.8	2.29	
Median (IQ range)	140.5 (101.5–191.3)	170.9 (119.7–229.7)	130.6 (96.7–171.4)	
Potassium (mmol/24 h urine)				
Mean (SD)	58.5 (22.3)	64.5 (24.8)	55.3 (20.1)	<0.001
S.E.	0.68	1.2	0.76	
Median (IQ range)	56.0 (43.0–71.0)	62.0 (47.0–78.0)	53 (41–66.5)	
NaCl intake (g/day)				
Mean (SD)	9.3 (4.2)	10.9 (4.5)	8.4 (3.7)	<0.001
S.E.	0.12	0.23	0.14	
Median (IQ range)	8.6 (6.2–11.7)	10.5 (7.3–14.0)	8.0 (5.8–10.5)	
Potassium intake (g/day)				
Mean (SD)	2.9 (1.1)	3.3 (1.2)	2.8 (1.0)	<0.001
S.E.	0.03	0.06	0.03	
Median (IQ range)	2.8 (2.1–3.5)	3.1 (2.3–3.9)	2.6 (2.0–3.3)	
Na-to-K ratio				
Mean (SD)	2.8 (1.4)	3.0 (1.6)	2.6 (1.2)	<0.001
S.E.	0.04	0.08	0.04	
Median (IQ range)	2.6 (1.8–3.5)	2.8 (1.9–3.8)	2.5 (1.8–3.3)	
Urinary iodine excretion (µg/L)	N = 577	N = 208	N = 369	
Mean (SD)	156.2 (142.19)	172.6 (144.9)	147.3 (139.9)	<0.001
S.E.	7.0	10.2	7.2	
Median (IQ range)	131.8 (95.4–174)	145.9 (108.58–191.6)	121.7 (91.4–167.1)	

**Table 3:** Distribution of participants’ iodine statuses according to the WHO criteria based on 24 h urinary iodine concentrations (µg/L).

Iodine Status	Iodine Concentration (µg/L)	Whole Group N = 577		Men N = 208		Women N = 369	
Insufficient		N	%	N	%	N	%
Severe	<20	2	0.3	1	0.5	1	0.3
Moderate	20–49	16	2.8	8	3.7	8	2.2
Mild	50–99	111	19.2	31	14.5	80	21.7
Adequate consumption	100–199	298	51.7	100	46.7	198	53.7
Above requirement	200–299	111	19.2	54	25.2	57	15.4
Excessive consumption	>300	39	6.8	14	6.5	25	6.8

**Table 4:** Differences in daily estimates of salt, potassium, and iodine intake between rural and urban areas and between the continental and Mediterranean parts of Croatia.

24 h Urine	Urban	Rural	*p*	Mediterranean	Continental	*p*
N	608	376		434	473	
NaCl intake (g/day)						
Mean (SD)	8.8 (3.9)	9.9 (4.4)	<0.001	8.0 (3.6)	10.3 (4.4)	<0.001
S.E.	0.16	0.23		0.17	0.23	
Median (IQ range)	8.3 (5.8–11.2)	9.4 (6.5–12.8)		7.4 (5.3–10.2)	9.9 (7.2–13.3)	
Potassium intake (g/day)						
Mean (SD)	3.0 (1.1)	2.8 (1.0)	0.006	3.0 (1.2)	2.8 (1.0)	<0.001
S.E.	0.04	0.09		0.05	0.04	
Median (IQ range)	2.9 (2.1–3.7)	2.7 (2.0–3.4)		2.9 (2.2–3.8)	2.6 (2.0–3.3)	
Na-to-K ratio						
Mean (SD)	2.6 (1.4)	3.0 (1.4)	<0.001	2.4 (1.4)	3.0 (1.3)	<0.001
S.E.	0.05	0.07		0.06	0.06	
Median (IQ range)	2.3 (1.6–3.1)	2.9 (2.1–3.7)		2.0 (1.5–2.9)	3.0 (2.3–3.8)	
Urinary iodine excretion (µg/L)	N = 183	N = 334		N = 219	N = 251	
Mean (SD)	168.9 (133.1)	146.4 (130.0)	0.007	145.8 (116.5)	161.5 (124.3)	0.012
S.E.	9.84	7.1		7.9	7.8	
Median (IQ range)	127.7 (103.2–193.2)	119.8 (92.6–109.2)		124.9 (90.3–171.2)	141.2 (103.9–183.0)	

**Table 5:** Daily urinary excretions of volume, sodium, potassium, and creatinine and estimates of salt, potassium, and iodine intake across different regions of Croatia.

	Continental			Coast	
	Central Part	North–East Part	North–West Part	North Part	South Part
N	143	234	154	102	434
24 h urine volume (mL)					
Mean (SD)	1631.3 (667.3)	1621.6 (652.5)	1515.7 (579.4)	1764.6 (840.2)	1544.3 (564.1)
S.E.	58.9	44.0	48.9	101.8	27.2
Median (IQ range)	1500.0 (1200–1972.5)	1560.0 (1152.5–1987.9)	1400.0 (1100.0–1800.0)	1500.0 (1180.0–2100.0)	1500.0 (1100.0–1900.0)
Urine creatinine (g/L)					
Mean (SD)	0.7 (0.3)	0.91 (0.414)	0.6 (0.3)	0.6 (0.3)	0.9 (0.4)
S.E.	0.02	0.02	0.02	0.03	0.02
Median (IQ range)	0.71 (0.52–0.93)	0.81 (0.61–1.11)	0.6 (0.42–0.87)	0.5 (0.42–0.79)	0.9 (0.6–1.2)
24 h sodium (dU)					
Mean (SD)	169.0 (69.8)	160.2 (75.3)	182.3 (66.7)	147.0 (63.4)	130.5 (59.6)
S.E.	6.1	5.1	5.6	7.6	2.8
Median (IQ range)	158.2 (119.7–216.3)	146.9 (109.0–204.0)	179.5 (133.3–220.8)	137.8 (98.8–185.0)	120.1 (87.0–163.2)
24 h potassium (dU)					
Mean (SD)	55.6 (20.1)	52.2 (20.0)	59.4 (18.8)	61.2 (21.4)	62.4 (25.0)
S.E.	1.7	1.3	1.5	2.5	1.2
Median (IQ range)	52.0 (41.0–66.8)	49.0 (38.0–66.0)	58.0 (46.0–71.7)	59.5 (47.0–74.5)	60.0 (45.0–76.0)
NaCl intake (g/day)					
Mean (SD)	10.3 (4.2)	9.8 (4.6)	11.1 (4.0)	9.0 (3.8)	8.0 (3.6)
S.E.	0.3	0.3	0.3	0.4	0.1
Median (IQ range)	9.7 (7.3–13.2)	9.0 (6.7–12.6)	11.0 (8.1–13.5)	8.4 (6.0–11.3)	7.3 (5.3–9.9)
Potassium intake (g/day)					
Mean (SD)	2.8 (1.0)	2.6 (1.0)	3.0 (0.9)	3.1 (1.0)	3.1 (1.2)
S.E.	0.09	0.06	0.08	0.13	0.06
Median (IQ range)	2.6 (2.1–3.4)	2.4 (1.9–3.3)	2.9 (2.3–3.6)	3.0 (2.3–3.7)	3.0 (2.3–3.8)
Na-to-K ratio					
Mean (SD)	3.1 (1.2)	3.2 (1.5)	3.1 (1.1)	2.4 (0.8)	2.3 (1.4)
S.E.	0.1	0.1	0.09	0.1	0.07
Median (IQ range)	2.9 (2.2–3.9)	2.9 (2.2–3.9)	3.1 (2.4–3.7)	2.3 (1.7–3.0)	2.0 (1.4–2.9)
Urinary iodine excretion (µg/L)	114	60	78	43	223
Mean (SD)	169.1 (164.4)	157.3 (83.5)	153.4 (64.2)	110.9 (147.3)	146.1 (136.7)
S.E.	15.5	10.7	7.3	22.4	9.2
Median (IQ range)	140.5 (105.8–181.2)	138.6 (99.9–196.1)	146.6 (111.3–179.5)	126.7 (79.0–169.3)	122.3 (90.4–171.0)

**Table 6:** CRASH and EH-UH 2 research plans.

1.	To determine the most important predictors (demographic, socioeconomic, etc.) of high salt and low potassium intake in men and women.
2.	To determine the most important predictors of high salt and low potassium intake, which will explain the observed differences between rural and urban areas and among different geographical regions.
3.	To determine the association between salt and potassium consumption with cardio–kidney–metabolic diseases and other poor lifestyle habits.
4.	To analyse the association between salt and potassium intake and BP.
5.	To analyse the association among salt reduction, the decrease in population BP, and the decrease in CV mortality in Croatia in the period from 2005 to 2022.
6.	To construct a model that will allow us to conclude how much additional iodine should be added per kilogram of salt if the salt intake were to be lowered by an additional 15% [66].

## Data Availability

The original contributions presented in the study are included in the article/Supplementary Materials, further inquiries can be directed to the corresponding author.

## Supplementary Materials

The following supporting information can be downloaded at: https://www.mdpi.com/article/10.3390/nu16162599/s1, Figure S1. Correlation between salt and potassium consumption in the whole group. Figure S2. Correlation between salt and potassium consumption in men. Figure S3. Correlation between salt and potassium consumption in women. Figure S4. Correlation between daily iodine excretion and estimated daily salt ingestion in the whole group. Figure S5. Correlation between daily iodine excretion and estimated daily salt ingestion in men. Figure S6. Correlation between daily iodine excretion and estimated daily salt ingestion in women.
