# Supplementary material for: Assessment of Salt, Potassium, and Iodine Intake in the Croatian Adult Population Using 24 h Urinary Collection: The EH-UH 2 Study
Source: Nutrients. 2024 Aug 7;16(16):2599. doi: 10.3390/nu16162599 (PMC11356790; doi:10.3390/nu16162599)
Supplement: Supplementary file 1 [file nutrients-16-02599-s001.zip › nutrients-3104907-supplementary.pdf]

Supplementary Materials

S1. Materials and Methods

*S1.1. **Study design and recruitment***

The participants selected via the randomisation list were informed about the project by telephone call from family physician and were included in the study according to the exclusion and inclusion criteria. Exclusion criteria for participation in the project were persons with terminal illness, dementia, paresis, amputation or immobilisation of a limb, acute illness, convalescent after surgery, pregnant women, lactating mothers, COVID - 19 infection within the last 3 months, those who were prescribed therapy with diuretics in the last two weeks prior to the urine sample collection date, and unsigned consent to participate in the research. The inclusion criteria were age over 18 years of age and a signed consent to participate in the research. In the case the invited subject had one or more exclusion criteria, the family physician contacted the next subject from the randomization list. For iodine status analysis, subjects with thyroid disorders or known consumption of iodine supplements were also excluded. After the participants had been included in the study, the nurses, members of the mobile examination team (MET) made an appointment for home visit. The home visit started with signing the consent to participate in the study. After signing informed consent, every participant was attributed a personal identification code to allow anonymization, and the first measurements of BP and heart rate were taken. Participants were given instruction on how to properly collect and handle urine during a 24-hour period and were instructed to fast for 12 hours prior to the examination at which blood samples would be taken. At the end of the home visits, the participants were invited for outpatient examinations.

S1.1.1. Questionnaire

The questionnaire (face-to-face interview) was used to collect data on participant's demographic (age, sex, place of residence), socio-economic status (SES), lifestyles (physical activity, smoking habit, alcohol consumption, diet, frequency of high salt food consumption, fruit and vegetable consumption, knowledge on dietary salt), personal and family history of cardio-kidney-metabolic diseases, and drug therapy. We used the same questionnaire which was used in our previous EH-UH 1 study [32].

S1.1.2. Anthropometry (physical measurements)

The outpatient examinations of the participants included anthropometric data measurements, measurement of brachial BP, central BP and arterial stiffness (pulse wave velocity, PWV), recording of electrocardiogram (ECG), and metabolic scale measurements followed by fasting blood drawing and collecting of morning spot urine sample. Few days before the outpatient examinations, participants were reminded to collect and bring 24-hour urine from the previous day. Office BP measurements were performed according to the European Society for Hypertension guidelines and recommendations of the Croatian Society of Hypertension [33,34]. BP was measured on both hands, and if there was a difference in BP over the left or right hand, as the higher value was taken as relevant, and further BP was measured on that arm. If there was no difference in BP between left and right arm, BP was measured on the non-dominant arm. BP and heart rate were measured using oscillometric device (OMRON M6 Comfort with smart cuff). The first measurement was discarded, and an average of the last two readings was used. Hypertension was defined as BP ≥ 140/90 mm Hg or taking medication for hypertension. The persons' weight and height were measured while they were clothed only in their underwear without shoes.

S1.2. Procedures of 24-hour urine collection

Sodium and potassium content in the urine were determined on the Abbott Alinity CC analyzer using the ion-selective electrode, indirect method after dilution of the sample (method of indirect potentiometry) (Alinity, Abbott, USA). The method is standardized according to same company NIST SRM 918 and NIST SRM 919 reference material. Calibration was performed every day before the start of work. Creatinine content was determined using the enzymatic method with creatininase on the same analyzer. Calibration

was performed once per lot of reagents using the same company calibrators traceable to the IDMS method and NIST reference material SRM 967 (substance creatinine purity of 99.7 ± 0.3%). Quality control data in 2018 were: accuracy 1,4% for sodium, 2,7% for potassium and 3,0% for creatinine, inter-assay variation coefficient 2,04% for sodium, 1,43% for potassium and 2,57 % for creatinine and intra-assay variation coefficient 1,79% for sodium, 1,32% for potassium, and 2,54 % for creatinine. In 2019 quality control dana were: accuracy 0,91% for sodium, 1,48% for potassium and 1,71% for creatinine, inter-assay variation coefficient 1,34% for sodium, 1,69% for potassium and 2,44 % for creatinine and intra-assay variation coefficient 1,28% for sodium, 1,42% for potassium and 2,07 % for creatinine. The respective data for the period 2021 were: accuracy 2,01% for sodium, 0,31% for potassium and 4,41% for creatinine, inter-assay variation coefficient 1,60% for sodium, 1,88% for potassium and 2,08% for creatinine and intra-assay variation coefficient 1,58% for sodium, 1,82% for potassium and 1,76 % for creatinine. Values for sodium or potassium (mmol/24 h) were converted into g/day (1 mmol = 0.023 g of sodium, 1 mmol = 0.039 g of potassium) to estimate dietary intake. The sodium value was multiplied by 2.5421 to convert dietary sodium intake into salt (NaCl) intake. Then, sodium values were multiplied by 1.05 (if approximately 95% of sodium ingested is excreted in the urine) [35]. Potassium dietary intake was calculated assuming 85% of the potassium ingested is excreted in the urine [36].

Urinary iodine was expressed in µg/l using multiplier 1.08 assuming 92% of iodine ingested is excreted in the urine. For determination of urinary iodine by modified microplate method, calibrators are prepared manually by dissolving KIO3 in deionized water. Working standards are 0, 5, 20, 40, 80, 120, 200, 400 and 1000 ug/L. Calibrators were used to re-evaluate lower detection limits at the end of standard curve and sometimes as internal control. Inter-assay coefficient of variation (n=15) is 9,0 % for low control value (arithmetic mean=170 ug/L), and 9,3 % for high control value (arithmetic mean=565), inter-assay CV=9,15%. Intra-assay coefficient of variation (n=45) is 15% for value on lower detection limit value (20 ug/L) and 10% on low control value (170 ug/L).

The cut-off values according to the WHO/EURO protocol were used [37]: < 5g/24-hour for salt consumption, >9 0 mmol/24-hour for potassium consumption, for iodine consumption: insufficient (<100 µg/l) with subcategories: severe (<20 µg/l), moderate (20–49 µg/l), mild (50–99 µg/l); adequate consumption (100–199 µg/l); consumption above requirement (200–229 µg/l); excessive consumption (≥ 300 µg/l) [38].

S2. Results

S2.3. Daily urinary excretions of creatinine, sodium, potassium, iodine, and estimated salt, potassium and iodine intake in the whole group

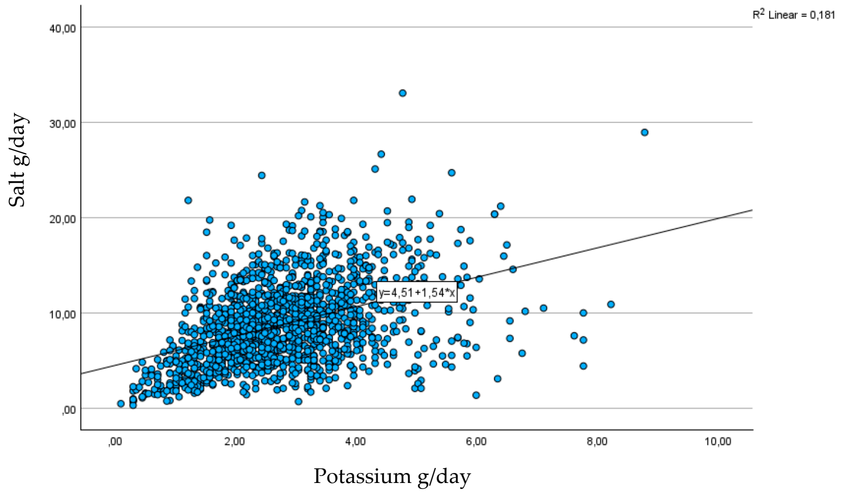

Figure S1. Correlation between salt and potassium consumption in the whole group

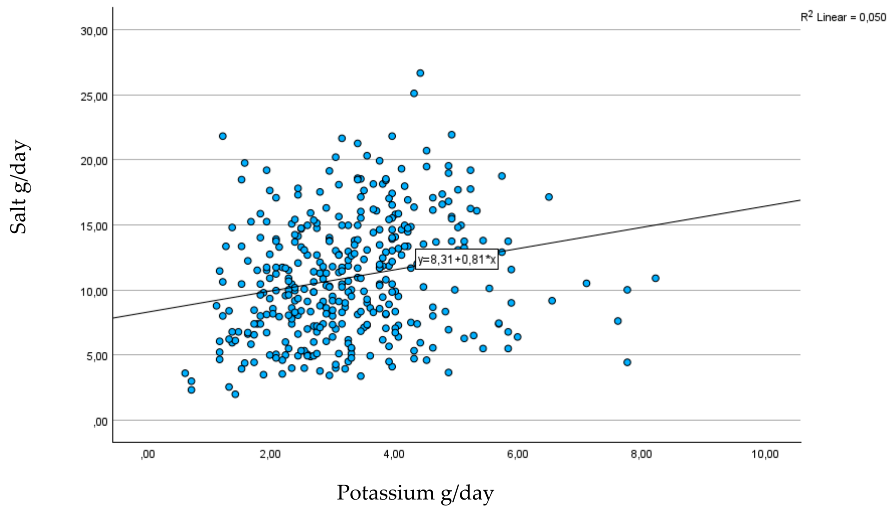

Figure S2. Correlation between salt and potassium consumption in men

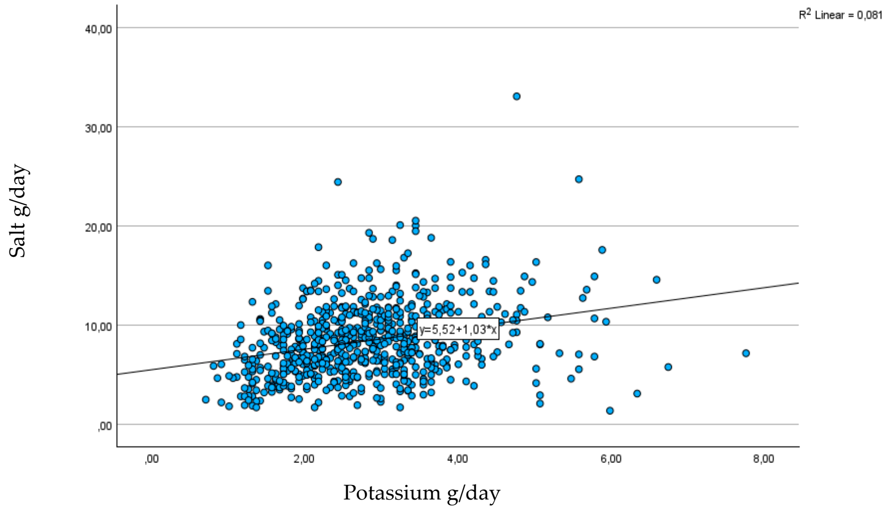

Figure S3. Correlation between salt and potassium consumption in women

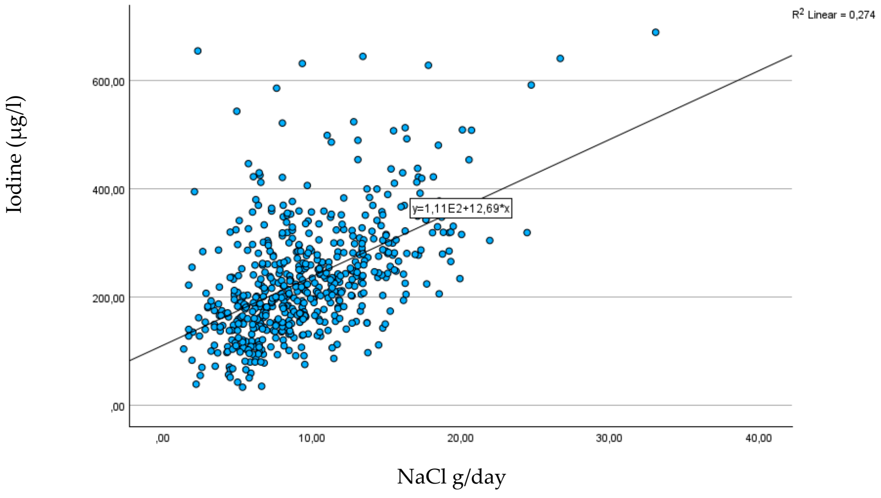

Figure S4. Correlation between daily iodine excretion and estimated daily salt ingestion in the whole group

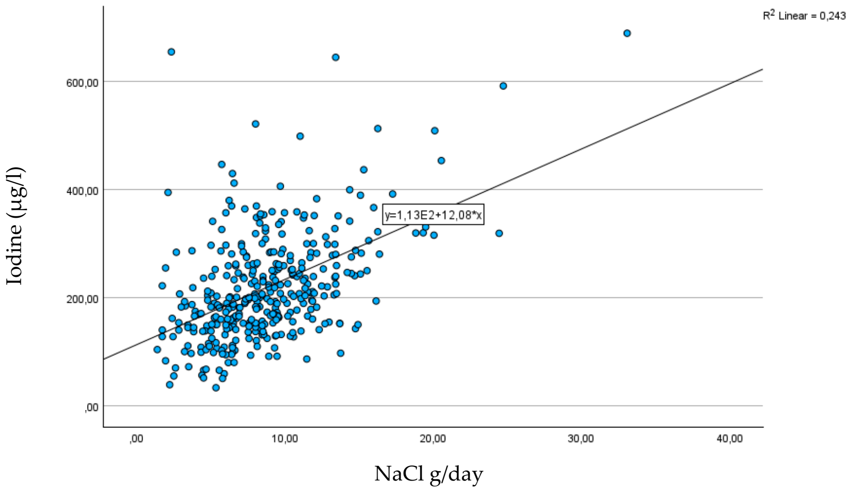

Figure S5. Correlation between daily iodine excretion and estimated daily salt ingestion in men

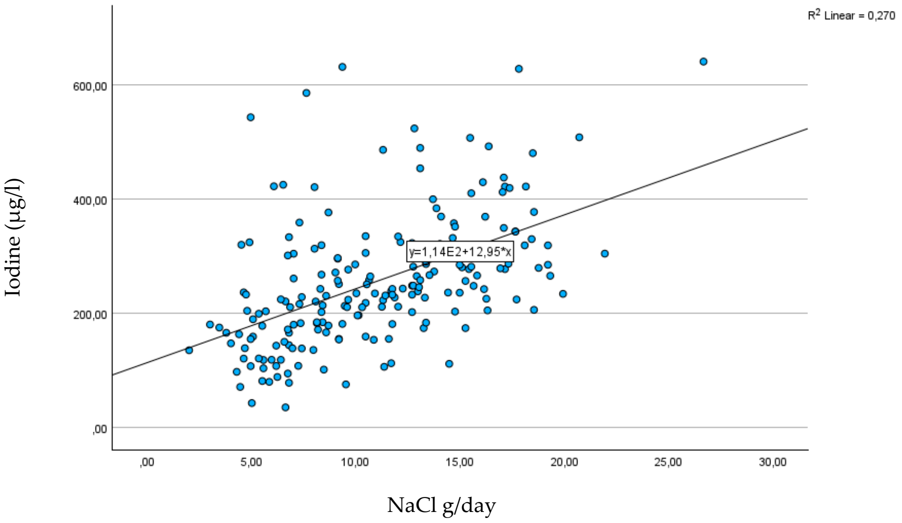

Figure S6. Correlation between daily iodine excretion and estimated daily salt ingestion in women
